# Small Molecule Inhibitors of Bcl-2 Family Proteins for Pancreatic Cancer Therapy

**DOI:** 10.3390/cancers3021527

**Published:** 2011-03-24

**Authors:** Ashiq Masood, Asfar S. Azmi, Ramzi M. Mohammad

**Affiliations:** 1 Department of Internal Medicine/Pathology, Karmanos Cancer Institute, Wayne State University, 4100 John R, HWCRC 732, Detroit, MI 48201, USA; E-Mail: amasood@med.wayne.edu; 2 Department of Pathology, Karmanos Cancer Institute, Wayne State University, 4100 John R, HWCRC 732, Detroit MI 48201, USA; E-Mail: azmia@karmanos.org; 3 Department of Oncology, Karmanos Cancer Institute, 4100 John R, HWCRC 732, Detroit, MI 48201, USA

**Keywords:** PC, Bcl-2 family proteins, small molecule inhibitors, apoptosis, cancer therapy

## Abstract

Pancreatic cancer (PC) has a complex etiology and displays a wide range of cellular escape pathways that allow it to resist different treatment modalities. Crucial signaling molecules that function downstream of the survival pathways, particularly at points where several of these pathways crosstalk, provide valuable targets for the development of novel anti-cancer drugs. Bcl-2 family member proteins are anti-apoptotic molecules that are known to be overexpressed in most cancers including PC. The anti-apoptotic machinery has been linked to the observed resistance developed to chemotherapy and radiation and therefore is important from the targeted drug development point of view. Over the past ten years, our group has extensively studied a series of small molecule inhibitors of Bcl-2 against PC and provide solid preclinical platform for testing such novel drugs in the clinic. This review examines the efficacy, potency, and function of several small molecule inhibitor drugs targeted to the Bcl-2 family of proteins and their preclinical progress against PC. This article further focuses on compounds that have been studied the most and also discusses the anti-cancer potential of newer class of Bcl-2 drugs.

## 1. Introduction

PC is a therapy refractory and deadly disease with a an annual mortality of ∼35,000 in the United States [1]. Amongst the mechanisms by which PC cells could escape any non-surgical therapy, anti-apoptotic protection seems to be the most relevant one [2]. Additionally, PC cells acquire resistance to apoptotic stimuli such as death ligands (FasL, TNF-related apoptosis inducing ligand (TRAIL)) or standard drugs (gemcitabine) by a great number of molecular alterations either disrupting an apoptosis inducing signal or counteracting the execution of apoptosis [3,4]. Among the other crucial pathway alterations observed in this resistant disease are deregulated Epidermal growth factor receptor pathway EGFR/MAPK/Ras/raf1- [5], PI3K/Akt- [6], TRAIL/TRAF2- or nuclear factor-κB IKK/NF-κB pathway [7] accompanied by deregulation in the expression of apoptosis regulators such as cIAP, Bcl-2, XIAP or survivin [8]. PC has been shown to overexpress Bcl-2 and its family members [9]. Therefore, blockade of Bcl-2 activity should become a novel therapeutic strategy for PC. To this end, many groups have been working to develop anticancer drugs that block the function of Bcl-2 members [10-12]. Drugs such as small-molecule inhibitor of Bcl-2, target multiple members of the Bcl-2 family and attenuate activation of Bcl-2. These drugs are designed to target the elongated groove of antiapoptotic proteins that normally bind the BH3 domain of proapoptotic effectors such as Bid, Bax, Bim, and others [12].

PC is a complex disease with a multitude of deregulated pathways. Median survival is four to six months and the five-year survival is less than 5% [11]. Standard chemotherapeutic agent gemcitabine or platinum-based genotoxic regimens such as oxaliplatin have little impact on improving the overall survival of PC patients [12]. Therefore, management of PC is an ongoing challenge and novel clinically-translatable therapeutic agents that can improve on the dismal survival statistics of PC are urgently needed. This proposal poses the critical question regarding the mechanism of drug failure in PC and addresses the problem by investigating a novel drug combination regimen. Although to date only partly understood, due to the heterogeneity of PC at the cell/tissue level, carcinogenesis progresses through the accumulation of genetic alterations resulting in a gain of cell growth and proliferation, and subsequently, in increased dissemination and metastatic potential [13]. Loss or gain of gene function may appear in the form of up-regulation of oncogenes, down-regulation of tumor suppressor genes, and deregulation of genomic maintenance/DNA repair genes, house-keeping genes, and genes that control the apoptosis/cell death/immortalization cascade [14-16]. PC arises from precursor lesions called pancreatic intraepithelial neoplasms (PanINs), which are characterized by the sequential accumulation of alterations in the *K-ras* oncogene and loss of the *CDKN2A*, p53, and/or *SMAD4* tumor suppressors along with upregulation of pro-survival Bcl-2 [17].

Although we know the frequencies of such mutations in PC, their specific functions during the development of PC remain unclear. PC is an oncogenic K-ras driven disease that has been shown to positively drive Bcl-2 expression that in turn can suppress other pro-apoptotic proteins such a PAR-4 [18]. This has showsn that Bcl-2 upregulation is among the most critically factors that crosstalk with other molecules to render PC therapy resistance [19,20].

It is well documented that Bcl-2 functions through heterodimerization with proapoptotic members of the Bcl-2 family to prevent mitochondrial pore formation and prevent cytochrome *c* release and initiation of apoptosis [13]. However, there is more evidence showing that Bcl-2 may play an oncogenic role through survival pathways other than its function at the mitochondrial membrane. It has been reported that Bcl-2 activates NF-κB by a signaling mechanism that involves Raf-1/MEKK-1–mediated activation of IKKβ [14]. Mortenson and colleagues have shown that overexpression of Bcl-2 increased the activity of AKT and IKK as well as NF-κB transcriptional activity in PC [15,16]. Kumar and colleagues found that Bcl-2–induced tumor cell proliferation and tumor cell invasion were significantly mediated by interleukin-8 [17]. Recently, Tucker and colleagues reported that Bcl-2 overexpression leading to maintenance of cyclin D1a expression may occur through p38 mitogen-activated protein kinase (MAPK)-mediated signaling pathways in human lymphoma cell lines [18]. Moreover, down-regulation of Bcl-2 also could modulate the expression of anhydrase IX (CAIX), vascular endothelial growth factor (VEGF), and pAkt in prostate cancer cell lines [19]. These studies provide evidence in support of the multi-functional role of Bcl-2 in cancer biology that is beyond its classical role in cell survival.

With respect to the multitude of anti-apoptotic pathways, a great number of molecular targets might be of high potential in novel therapy strategies, which is the theme of this issue. Even though these early studies encouraged an application in a clinical setting, most of the trials have been rather disappointing to date. Thus, new molecular targets and novel concepts of combination therapies need to gain access into clinical trials—either in neoadjuvant/adjuvant or in palliative treatments. Apoptosis (also known as programmed cell death) is a natural, active and tightly controlled form of cell death through which multi-cellular organisms get rid of damaged and aging cells. It is also deliberated a stress induced process of cellular communication [21]. There are two major apoptotic signaling pathways, *i.e.*, the death receptor (extrinsic) pathway and the mitochondria (intrinsic) pathway [22]. In addition, there is another pathway that in that involves T-cell mediated cytotoxicity and perforin-granzyme-dependent killing of the cell. While granzyme B and granzyme A proteases are responsible for inducing cell death in this pathway [23].

**Figure 1.**
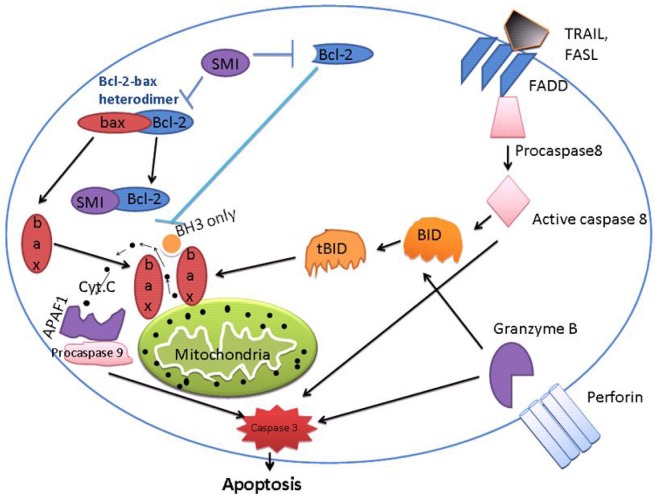
The Apoptotic Process. There are two major apoptotic signaling pathways: the extrinsic pathway and the mitochondria (intrinsic) pathway. In addition, there is another pathway that involves T-cell mediated cytotoxicity and perforin-granzyme-dependent killing of the cell. The mitochondrial (intrinsic) pathway is regulated by Bcl-2 family and activated by mitochondrial disruption with subsequent cytochrome c release. Initiators of this pathway include UV irradiation and cytotoxic drugs. An ‘apoptosome’ is formed by the interaction of cytochrome c, Apaf-1, d-ATP/ ATP and procaspase-9 with subsequent initiation of the caspase cascade.

These intrinsic, extrinsic, and granzyme B have different modes of initiation but have the same outcome: they lead to activation of a cascade of proteolytic enzymes, members of caspase family [24] (Figure 1). Granzyme A, a serine protease, causes cell death by DNA damage by single-stranded nicks, independent of caspases [25]. The mitochondrial (intrinsic) pathway is regulated by the BCL-2 family and activated by mitochondrial disruption with subsequent cytochrome c release. Initiators of this pathway include UV irradiation and cytotoxic drugs. An ‘apoptosome’ is formed by the interaction of cytochrome c, Apaf-1, d-ATP/ ATP and procaspase-9 with subsequent initiation of the caspase cascade [26]. Overexpression of BCL-2 and associated anti-apoptotic proteins Bcl-xL, Mcl-1, and BCL-W occurs in substantial subsets of common cancer types that include pancreatic, ovarian, lymphoma, multiple myeloma, lung adenocarcinoma, prostate adenocarcinoma, *etc* [27,28]. These Bcl-2 proteins can essentially make cancer cells resistant to a variety of chemotherapeutic agents and therefore these proteins are currently important targets for the development of new anti-cancer agents [29].

## 2. Bcl-2 Family of Proteins

Bcl-2, the founding member, was identified more than 20 years ago at the chromosomal breakpoint of t (14; 18) (q32; q21) lymphomas. Bcl-2 supports neoplastic growth, not only by stimulating cellular proliferation, but rather by blocking cell death [30-32]. More Bcl proteins were identified since then and there are at least 25 members identified so far [33]. Bcl-2 family members are grouped into three classes based on the number of BH (Bcl-2 Homology) domains they share. 1. Anti-apoptotic: Bcl-2, Bcl-xL, Bcl- W, Mcl-1, Bcl-B or BCL 2L10 and Bcl-A1 or Bcl -2A1 proteins possess four BH domains -BH1-4; 2. Pro-apoptotic: Bax, Bak and Bok or MTD; 3. BH-3 only proteins (Bad, Bik, Bid, Hrk, Bim, Bmf, Noxa, Puma).

Both apoptotic and BH-3 proteins are characterized by presence of only the BH3 domain [34,35]. The intrinsic (mitochondrial) apoptotic pathway is controlled by the balance between anti-apoptotic proteins belonging to the Bcl-2 family and pro-apoptotic proteins bearing a single BH3 domain as mentioned above [36]. BH-3 proteins are responsible for triggering apoptosis in response to various cellular stresses as mentioned above. In healthy cells, pro-apoptotic proteins, Bax, Bid and Bad reside in the cytosol. On initiation of apoptosis, these pro-apoptotic proteins translocate to the outer-mitochondrial membrane, causing the mitochondria to lose membrane potential. Recent studies also showed, Bak molecules present on mitochondria are activated on receiving apoptotic signals. On activation, Bak exposes its BH-3 domain,allowing dimerization by insertion of the BH-3 domain of one Bak protein into the hydrophobic groove of another. The symmetric Bak dimers formed after BH3:groove interaction is postulated to contain additional interaction interfaces that lead to more advanced order oligomers. The result is homo-oligomerization and permeabilization of the outer mitochondrial membrane (MOMP). A similar step of activation is also suggested in Bax [37].

Many investigators believe mitochondrial permeablization to be a “point of no return”. The reasons behind this statement are few. Following permeablization of mitochondrial membrane, cytochrome c is released from mitochondria; it forms an apoptosome as explained above which lead to activation of caspases in the cytosol and then final accomplishment of cellular structure degradation. Permeablization of the mitochondrial membrane also leads to mitochondrial dysfunction along with a fall in ATP or pro-apoptotic factors (AIF, endonuclease) release that causes caspase-independent cell death [38]. From the above discussion, it is clear that MOMP is a greatly organized process, principally controlled through interactions between pro- and anti-apoptotic members of the B cell lymphoma 2 (Bcl-2) family [39].

### 2.1. Earlier Therapies Targeting Bcl-2 Proteins

Bcl- 2 anti-sense therapy was an initial advancement towards this goal of targeting Bcl-2 to inhibit its overexpression [40-42]. Among different Bcl-2 anti-sense drugs Oblimersen sodium showed promise. Oblimersen specifically binds to the first six codons of the human Bcl-2 mRNA sequence, causing degradation of *Bcl-2* mRNA, resulting in consequent reduction in Bcl-2 protein translation and intracellular concentration [43]. Oblimersen was used in a number of clinical trials for different malignancies [44-50]. However, a phase III trial in melanoma did not show a survival benefit. Therefore, it was not approved by FDA. However, a number of other trials are underway [51] Some Bcl_XL_ antisense strategies were tested against PC in the early part of this decade where Xu and co-workers showed that Bcl_XL_ antisense oligonucleotides can suppress pancreatic tumor growth and also sensitize these cells to gemcitabine [52]. Nevertheless, this remains among the only successful study for antisense strategy against PC.

Another approach would be developing an antibody that would block activity of Bcl-2. An intracellular anti-Bcl-2 single chain antibody has been shown to increase drug-induced cytotoxicity in the MCF-7 breast cancer cell line as well as other cancers [53]. Moreover, other fascinating approaches include a ribozyme 200 against Bcl-2 and also synthetic cell permeable Bak BH-3 peptide, that was partially successful both *in vitro* and *in vivo* against myeloid leukemia growth [54]. These approaches have a number of limitations that include short half-life of anti-sense therapies due to rapid enzymatic degradation. Furthermore, lack of success of anti-sense compounds in a number of studies has reduced interest among scientists. However, second and third generation compounds have been developed and may bring back enthusiasm again [55]. Similarly, issues occurred with antibody, ribozymes or peptides therapies, such as lack of stability and effective delivery. Researchers developed another approach in which a biochemical strategy was used to make ‘stabilized a-helix of Bcl-2 domains’ (SAHBs) by hydrocarbon stabling. These BH3 peptides are helical, proteoresistant, cell permeable, and have high affinity to multi-domain member pockets [56-59].

## 3. Small Molecule Inhibitors of Bcl-2 Proteins and Their Progress against Pancreatic Cancer

From the above discussion, it is clear that none of therapy has been proven to be helpful when it comes to taking these drugs from the laboratory to the clinic. The last 20 years has witnessed enormous information in delineating the protein machinery responsible for apoptosis. It is widely believed that the equilibrium (rheostat model) between pro-apoptotic and apoptotic proteins determine whether a cell undergoes apoptosis or not [60-63]. The three dimensional structure of Bcl-xL disclosed a hydrophobic groove into which Bim or BID domains are able to bind [64]. This binding is essential for anti-apoptotic pathway function. Hypothetically, SMIs bind to the hydrophobic groove of Bcl-2, which will block the hetrodimerization of Bcl-2 with pro-apoptotic members of the Bcl-2 protein family, such as Bid and Bim. Drug occupation of the hydrophobic groove is thus thought to neutralize the anti-apoptotic function of Bcl-2 (and others) and induce apoptosis. There are multiple SMIs available that have shown great promise. In this review we will discuss a few of these specifically for their preclinical efficacy against pancreatic cancer.

### 3.1. Gossypol (BL 193)

Gossypol is a natural compound that was extracted from cotton seed in 1915 [65]. However, it was extensively studied as contraceptive and anti-cancer drug since the 1980s [66-69]. It is chemically reactive due to its six phenolic hydroxyl groups and two aldehydic groups [70]. Natural gossypol occurs in racemic form and levo isoform is currently in clinical trials [71] Gossypol was first used in study of glial tumors but its mechanism of action was unknown at that time [72]. The (−)-BL-193 (levo isoform) has been shown to be more potent than either isoforms in its growth-inhibitory effects. Multidimensional nuclear magnetic resonance methods have shown (−)-BL-193 binds the hydrophobic groove of Bcl-2 and Bcl-xL [73]. Gossypol has anti-cancer activity due to widespread effects on cells that include regulating Bcl-2 proteins and caspases [74], DNA damaging capacity [75, 76], activating p53, [77] ability to generate ROS and cytochrome c release [78]. Gossypol is also seen to enhance TRAIL-induced apoptosis by upregulation of TRAIL death receptors through the ROS-ERK-CHOP-DR5 pathway in colon cancer cells [79]. It has also been shown to induce beclin-1-dependent and – independent autophagic response in breast cancer cells, but autophagy was cytoprotective contrary to belief that it may aid in apoptosis. Gossypol is currently in pre-clinical testing.

Our group has investigated the effect of gossypol on PC growth in multiple cell lines. (-)-Gossypol showed a concentration-dependent growth inhibition effect against BxPC-3 PC cell line and induced apoptosis with no effect on normal peripheral blood lymphocytes. Results from co-immunoprecipitation experiments indicate that the effect of (-)-gossypol is mediated, at least in part, via disrupting the heterodimerization of Bcl-xL with Bim in BxPC-3 PC cells. (-)-Gossypol completely disrupts Bcl-xL/Bim heterodimerization with no change in the total Bcl-xL or Bim protein, indicating that (-)-gossypol treatment does not affect the levels of Bcl-xL and Bim proteins. In addition, the combination of (-)-gossypol with genistein showed significantly greater growth inhibition compared with either agent alone [80].

### 3.2. TW-37

TW-37 is a benzenesulfonyl derivative, second generation SMI, derived from Gossypol [81]. Our laboratory has extensively studied TW-37 for its action in leukemia, lymphoma and PCs [82-84]. In addition to its anti-apoptotic activity, it was also shown to have anti-angiogenic activity [85]. Like other SMI's, it was originally developed to target the BH-3 binding groove in Bcl-xL. We have previously shown that TW-37 has high affinity for Bcl-2 in addition to Bcl-xL and targets Mcl-1 unlike other SMIs. This unique feature of TW-37 to block Mcl-1 is of great significance as Mcl-1 is emerging as a key participant in the pro-survival machinery. In our laboratory, we have also shown that TW-37 induces apoptosis in PCs through a novel NOTCH-1 pathway [87].

TW-37 is perhaps the most studied Bcl-2 small molecule inhibitor against PC. Using multiple cellular and molecular approaches we found that TW-37, in nanomolar concentrations, inhibited PC cell growth in a dose- and time-dependent manner. This was accompanied by increased apoptosis and concomitant attenuation of NF-kappaB, and downregulation of NF-kappaB downstream genes such as MMP-9 and VEGF, resulting in the inhibition of PC cell migration, invasion and angiogenesis *in vitro* and antitumor activity *in vivo*. From these results, it was concluded that TW-37 is a potent inhibitor of progression of PC cells, which could be due to attenuation of Bcl-2 cellular signaling processes. Our findings provided evidence that TW-37 could act as a small-molecule Bcl-2 inhibitor on well-characterized PC cells in culture as well as when grown as tumor in a xenograft model. We also suggest that TW-37 could be further developed as a potential therapeutic agent for the treatment of PC.

We also showed the TW-37 and another inhibitor ApoG2 could induce PC growth inhibition through the novel apoptosis protein Par-4. Sensitivity to apoptosis was directly related to the expression levels of PAR-4 (R = 0.92 and R2 = 0.95). Conversely, small interfering RNA against PAR-4 blocked apoptosis, confirming that PAR-4 is a key player in the apoptotic process. In combination studies with gemcitabine, pretreatment with SMI led to sensitization of Colo-357 cells to the growth-inhibitory and apoptotic action of a therapeutic drug, gemcitabine. Our results suggested that the observed antitumor activity of both ApoG2 and TW-37 was mediated through a novel pathway involving induction of PAR-4. To our knowledge, these were the first studies reporting SMI-mediated apoptosis involving PAR-4 in PC [84].

In another study while investigating the true mechanism of action of the Bcl-2 inhibitor TW-37, we found that TW-37 induces cell growth inhibition and S-phase cell cycle arrest, with regulation of several important cell cycle-related genes like p27, p57, E2F-1, cdc25A, CDK4, cyclin A, cyclin D1, and cyclin E. The cell growth inhibition was accompanied by increased apoptosis with concomitant attenuation of Notch-1, Jagged-1, and its downstream genes such as Hes-1 *in vitro* and *in vivo*. We also found that down-regulation of Notch-1 by small interfering RNA or gamma-secretase inhibitors before TW-37 treatment resulted in enhanced cell growth inhibition and apoptosis. Our data suggest that the observed antitumor activity of TW-37 is mediated through a novel pathway involving inactivation of Notch-1 and Jagged-1 [87].

### 3.3. Apogossypolone (ApoG2)

ApoG2 is a third generation SMI that has also been studied extensively in our laboratory. It was developed to decrease reactivity and toxicity of gossypol. It was chemically made by the removal of two reactive aldehyde groups on poly-phenolic rings of gossypol. We have shown that ApoG2 blocks binding of Bcl-2 and Bim and induces apoptosis in lymphoma cell lines with minimal toxicity [88]. In addition, APO G2 has shown significant activity against follicular small cleaved lymphoma, pre-B cell acute lymphoblastic leukemia, multiple myeloma, mantle cell leukemia, prostatic cancer, and PC [89-93].

Our studies showed that when ApoG2 was combined with gemcitabine, increased cytotoxicity and apoptosis was evident. Co-immunoprecipitation experiment revealed that ApoG2 blocks the heterodimerization of Mcl-1/Bax and Bcl-2/Bim in cells. Furthermore, administration of ApoG2 with gemcitabine resulted in a statistically higher antitumor activity compared with either ApoG2 or gemcitabine alone in a severe combined immunodeficiency mouse xenograft model. These studies concluded that ApoG2, which functions as a potent pan-Bcl-2 family inhibitor, seems therapeutically promising for future translational studies including the treatment of PC.

**Figure 2.**
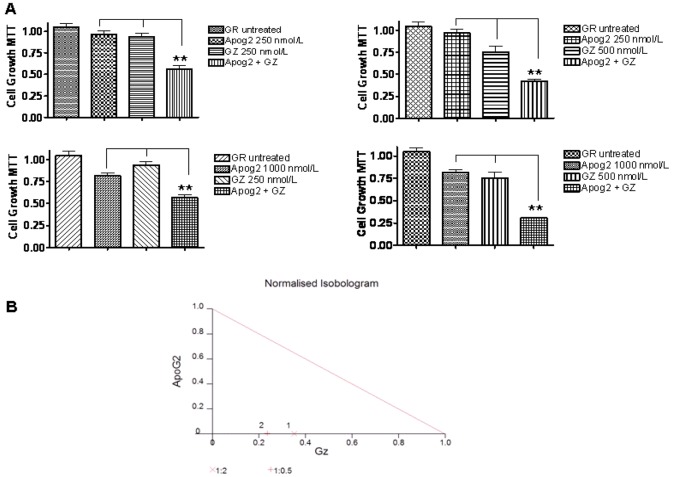
Bcl-2 family inhibitor ApoG2 synergizes with gemcitabine. (**A**) ApoG2 synergizes with gemcitabine and induces superior growth inhibition compared to single agents in gemcitabine resistant PC cells (MiaPaCa-GR). MiaPaCa-GR cells were developed by growing MiaPaCa-2 cells in 100 nm gemcitabine-containing media for 4 weeks. The cells were exposed to the indicated concentrations of (top and bottom panels) ApoG2 (250 nmol/L and 1000 nmol/L); gemcitabine (250 nmol/L; 500 nmol/L) or their respective combinations for 72 hrs (multiple concentrations were used to obtain statistically relevant and Isobologram analysis eligible data). Growth inhibition was analyzed by MTT assay. Note the combination has greater growth inhibition than single agent treatment. (**B**) Isobologram analysis for synergistic interaction using Calcusyn software. Combination index <1 means synergy. ** p<0.01 single agent *vs.* combination treatment. At similar doses, ApoG2 does not induce growth inhibition or apoptosis in normal human pancreatic ductal epithelial cells (HPDE).

We are further investigating the combination of ApoG2 and gemcitabine in a gemcitabine resistant cell line MiaPaCa-GR. Our ongoing studies in PC with ApoG2 show that this is among the most potent Bcl-2 SMI developed to date. ApoG2 not only shows higher efficacy against PC that TW-37 or AT-101 it also shows superior synergistic effects when combined with gemcitabine. Most interestingly, these studies were performed against a highly resistance PC cell line MiaPaCa that was made resistant by continuous exposure to gemcitabine. The drug synergized effectively with gemcitabine with a combination index value less than 1 (CI < 1) (Figure 2). Further *in vivo* studies in different xenografts and orthotopic animal models are underway. Some of our pancreatic cancer studies using Bcl-2 inhibitors are summarized in Table 1.

### 3.4. AT-101

It is an oral, pan Bcl-2 inhibitor [96], chemically (-)-gossypol, with the trade name AT-101. Along with topotecan, it is currently in multiple phase I/II trials in refractory small cell lung cancer [97], phase II clinical trials are ongoing in CLL (in combination with rituximab) and in hormone refractory prostate cancer (in combination with docetaxel) [98]. Furthermore, AT-101 has also shown activity against multiple myeloma cells [99,100].

### 3.5. ABT 737

This SMI was developed by Abbott laboratories using structure-activity relationship (SAR) by NMR strategy. ABT-737 mimics the BH-3 domain of BAD and binds selectively Bcl-2, Bcl-xL and Bcl-w but it binds with poor affinity to Mcl-1 and Bfl-1 [101]. In addition, ABT737 directly activates Bax or Bak and releases cytochrome c from mitochondria *in vitro* [102]. ABT-737, when used alone in different cancer cells, showed up regulation of Mcl-1 protein expression [103]. This problem was solved by many authors by using combination regimens [104-107]. ABT 269, an oral form of ABT 737 is in clinical trials [108]. It inhibits anti-apoptotic proteins Bcl-2, Bcl-xL and Bcl-w, and unlike ABT 737, has shown single agent efficacy in numerous small cell lung carcinoma (SCLC) and leukemia/lymphoma cell lines [109-111]. In addition, recent study had shown its potentiating effect in combination with common chemotherapeutic agents and regimens (VAP, CHOP, R-CHOP, Rituximib, etopside, *etc.*) in B-cell lymphoma and multiple myeloma. It was also seen that Bortezomib strongly synergized with ABT-263 in mantle cell lymphoma [112]. The most common toxicity observed of this agent is thrombocytopenia [113]. Additionally, Abbott laboratories using SAR by NMR and structure based drug design have recently developed a drug that is a highly potent, selective, anti-cancer agent that would potentially overcome Bcl-x_L_ mediated thrombocytopenia observed with ABT-263 [114].

Recently Sinicrope and colleagues have shown that ABT-737 can synergistically enhance TRAIL-mediated cytotoxicity in human PC cell lines. ABT-737 was shown to enhance TRAIL-induced apoptosis as shown by DNA fragmentation, activation of caspase-8 and Bid, and cleavage of caspase-3 and poly(ADP-ribose) polymerase. Mechanistically, Bax conformational change induced by TRAIL was enhanced by ABT-737. ABT-737 further disrupted the interaction of Bak with Bcl-xL while Bim small hairpin RNA (shRNA) was shown to attenuate caspase-3 cleavage and to reduce the cytotoxic effects of TRAIL plus ABT-737. The authors also noted that Mcl-1 shRNA potentiated caspase-3 cleavage by ABT-737 and enhanced its cytotoxic effects. Taken together these studies confirmed that ABT-737 could augment TRAIL-induced cell killing by unsequestering Bim and Bak and enhancing a Bax conformational change induced by TRAIL. These findings suggest a novel strategy to enhance cross-talk between the extrinsic and intrinsic apoptotic pathways to improve therapeutic efficacy against PC.

### 3.6. Obatoclax (GX-015-070)

Obatoclax (GX15-070) is a small-molecule indole bipyrrole compound that antagonizes Bcl-2, Bcl-xL, Bcl-W and Mcl-1 [115]. Obatoclax was discovered after researchers learned that Bcl proteins have potential to undergo conformational changes and they used high throughput screening of natural compound libraries that upset protein-protein interactions [102]. Obatoclax has the ability to inhibit the direct interaction between Mcl-1 and Bak and was seen to overcome the resistance to ABT-737 and the proteasome inhibitor bortezomib [116]. Obatoclax can trigger apoptosis in NSCLC cells and can enhance cisplatin based chemotherapy-induced death [117]. In a preclinical study, obatoclax was seen to induce potent cytotoxic responses against myeloma cells and in addition, enhanced the antimyeloma activity induced by melphalan, dexamethasone, or bortezomib [118]. Lately, obatoclax has been shown to induce Bax-mediated apoptosis in cholangiocarcinoma [119]. Moreover, this compound has shown activity against a wide variety of cancer cells that include mantle cell lymphoma, esophageal cancer cells, melanoma and PC cells [120-123].

Obatoclax has been tested for its potency against PC in combination with TRAIL. Obatoclax reduced the viability of PANC-1 and BxPC-3 cell lines and synergistically enhanced TRAIL-mediated cytotoxicity. Obatoclax enhanced TRAIL-mediated apoptosis, as shown by Annexin V labeling, which was accompanied by caspase activation (caspase-8, -9, and -3) and cleavage of Bid. Obatoclax potentiated TRAIL-mediated Bax/Bak activation and the release of mitochondrial cytochrome c, Smac, and AIF. Mechanisms underlying the apoptotic effect of obatoclax included displacement of Bak from its sequestration by Bcl-xL or Mcl-1 and release of Bim from Bcl-2 or Mcl-1. Bid knockdown by short hairpin RNA attenuated caspase cleavage and cytotoxicity of obatoclax plus TRAIL. Bim knockdown failed to inhibit the cytotoxic effect of obatoclax alone or combined with TRAIL, yet attenuated TRAIL-mediated cytotoxicity. AIF knockdown attenuated cytotoxicity of the drug combination. These studies concluded that obatoclax potentiates TRAIL-mediated apoptosis by unsequestering Bak and Bim from Bcl-2/Bcl-x(L) or Mcl-1 proteins and this drug combination enhances Bid-mediated crosstalk between the mitochondrial and death receptor-mediated apoptotic pathways and may represent a novel therapeutic strategy against PC.

### 3.7. HA 14-1

HA14-1 (ethyl 2-amino-6-bromo-4-(1-cyano-2-ethoxy-2-oxoethyl)-4H-chromene-3-carboxylate) ligand of Bcl-2 surface pocket was discovered by Wang *et al.* [124]. This molecule has shown activity on a number of cancer cell lines and enhances cytotoxicity of a number of cancer cell lines [125-127]. However, recent study showed that HA 14-1 is unstable at physiological conditions. It decomposes rapidly and generates ROS that could be mediators of cell death and this compound should be used cautiously as a qualified antagonist against antiapoptotic Bcl-2 proteins [128]. However, s-HA 14-1 was developed that did not induce ROS formation.

### 3.8. Toxicity Related Issues of Bcl-2 Inhibitors

Despite tremendous advances in the development of Bcl-2 inhibitors over the last 10 years their clinical progress has been very slow. Although many such molecules have been synthesized, rigorous verification of their specificity has often been lacking. Further studies have revealed that many putative Bcl-2 inhibitors are not specific and have other cellular targets, resulting in non-mechanism based toxicity. As yet the specificity of other agents, such as obatoclax and TW-37, to kill Bax/Bak null fibroblasts has not been definitively ascertained, although some reports indicate that obatoclax may be active in Bax/Bak null fibroblasts [129]. These studies suggest that a variety of Bcl-2 antagonists have additional targets besides inhibiting antiapoptotic Bcl-2 family members and these additional targets may lead to unpredicted, non-mechanism based toxicity. The notion that it is necessary to neutralize both arms of the antiapoptotic Bcl-2 family raises important questions with regard to the specificity of the Bcl-2 inhibitors. Researchers are diligently working in the direction of developing a pan Bcl-2 inhibitor that can additionally block or suppress Mcl-1 and Bcl2A1. Obatoclax is a pan Bcl-2 inhibitor whereas ABT-737 and ABT-263 are both Bad-like BH3 mimetics, which only inhibit Bcl-2, Bcl-X_L_ and Bcl-w but do not inhibit Mcl-1 and Bcl2A1 [116]. Resistance to ABT-737 has been linked to high expression levels of Mcl-1 and in many instances this resistance can be overcome by treatment with an agent(s) that decreases Mcl-1, such as seliciclib, a cyclin-dependent kinase inhibitor [130]. In contrast, obatoclax overcomes Mcl-1 mediated resistance to apoptosis partly by interfering with Mcl-1–Bak interactions [116]. Thus, solely from an efficacy viewpoint, it would be preferable to use a pan rather than a more specific Bcl-2 inhibitor. However, issues of toxicity, both mechanism and non-mechanism based, have also to be considered. Owing to limited *in vivo* studies to date on most of the Bcl-2 inhibitors, relatively little is known about their potential toxicities, with the possible exceptions of ABT-737 and gossypol. ABT-737 is generally well tolerated *in vivo* but has been reported to cause a concentration-dependent and rapid decrease in circulating platelets and lymphocytes without affecting platelet aggregation [131]. ABT-737, when tested in dogs, rapidly decreases platelets that return to baseline within three days and is compatible with a mechanism involving platelet destruction rather than a mechanism involving decreased production from megakaryocytes as observed following conventional cytotoxic chemotherapy. As Bcl-X_L_ is critical in limiting the proapoptotic activity of Bak in platelets, the observed thrombocytopenia appears to be because of inhibition of Bcl-X_L_ resulting in activation of Bak [131]. Older platelets contain less Bcl-X_L_ than younger platelets and are more susceptible to ABT-737, which helps to explain the almost full recovery of platelets in mice dosed daily with ABT-737. However, it should be noted that non specificity can also be beneficial in certain cases. For example, our group has shown that both ApoG2 and TW-37 can target Par-4 activation that leads to cancer cell specific killing [84]. This is an example where non specificity of these inhibitors benefits their overall activity. Nevertheless, the more specific the inhibitor for individual Bcl-2 family members, the less non-mechanism based toxicity would be expected. Pan Bcl-2 inhibitors are more likely to exhibit mechanism-based toxicities than more specific inhibitors. For example, a pan Bcl-2 family inhibitor that inhibits Mcl-1 may induce toxicities in cells or tissues where there is an important constitutive function of Mcl-1.

### 3.9. Future Directions

The most potent and clinically acceptable Bcl-2 inhibitor AT-101 is currently in 20 different clinical trials around the globe (ClinicalTrials.Gov). The agent has shown immense promise as a single agent and has been shown to synergize with different standard chemotherapeutic drugs. In Phase I and Phase II trials, AT-101 has demonstrated single-agent cytoreductive activity in several cancers, including chronic lymphocytic leukemia (CLL), non-Hodgkins lymphoma (NHL), and prostate cancer. Phase II combination trials were conducted in several cancers, including hormone-refractory prostate cancer and non-small cell lung cancer (with Taxotere® [docetaxel]), B-cell malignancies (with Rituxan® [rituximab]), small cell lung cancer (with Hycamtin® [topotecan]), glioma (with Temodar® [temozolomide], +/− radiotherapy) and esophageal cancer (with docetaxel, 5-fluorouracil and radiotherapy). Clinical trials are ongoing in the US and Europe and Ascenta is collaborating with Ascenta Pharma Group Corporation (located in Hong Kong and Shanghai) for the clinical development of AT-101 in China. Even though AT-101 is in so many different clinical trials, however, it is still far from a specific Bcl-2 inhibitor. Future research will focus on developing a more potent inhibitor with better specificity against Bcl-2 family proteins. Due to the dynamics of Bcl-2 protein structure, chemistry, mode of binding and efficacy, it is not an easy task to develop a perfect inhibitor. For example, both TW-37 and ApoG2 showed immense potency in the preclinical laboratory setting, however, they have not advanced to the clinic due to pharmacokinetic and toxicity related issues.

Over the years different groups have shown that Bcl-2 inhibitors can target non Bcl-2 targets as well. Therefore, in order to delineate the entire set of pathways modulated by these inhibitors our laboratory is pursuing a systems biology approach (on AT-101) to obtain drug target gene signatures of these. This will help in understanding the mechanism of action of these inhibitors and aid in the rational design of single agent and combination regimens in genetically pre-defined subset of cancer patients. Other approaches include nano-encapsulation of Bcl-2 inhibitors to increase bioavailability and tumor targeted combinations with vaso-active peptide receptor engrafted sterically stabilized Bcl-2 inhibitor micelle formulations.

## 4. Conclusions

PC is a deadly disease that is considered incurable. The dismal survival rate points towards the urgent need for the development of newer drugs against this malignancy. Deregulation of apoptotic machinery in PC has been acknowledged as a major contributor to the observed resistance to chemotherapy. Therefore, agents that target the apoptotic machinery leading to PC cell death may offer a better strategy against this disease. Over the last two decades the advancement of our understanding of apoptosis and the recognition of its key players has led to development of various strategies to selectively destroy cancer cells. Initial strategy to target anti-apoptotic protein translation by using antisense technology did not bring much success. However, small molecule inhibitors have shown promise against PC and have brought back enthusiasm among researchers. Small molecule inhibitors of Bcl-2 family proteins are specific and targeted agents that can be given orally and do not have any appreciable toxicity. This new class of drugs is increasingly being realized as effective anticancer agents either alone or to sensitize cancer cells to standard chemotherapeutics agents. Their emerging potential as enhancers of chemotherapeutic drugs can be a significant development that can be used as a future strategy against deadly tumors. We believe that in the next decade or so, as more key regulators of apoptosis are uncovered, new SMIs will continue to emerge to target these apoptotic proteins and will significantly improve the PC care.

## Figures and Tables

**Table 1.** Pancreatic cancer studies using Bcl-2 inhibitors.

**Bcl-2 Inhibitor**	**Study**	**Reference**
Gossypol	Inhibition of Bcl-2/Bcl_XL_ in BxPC-3 pancreatic cancer cell line	[1]
TW-37	Inhibition of pancreatic cancer growth and invasion	[81]
TW37	Inhibits Notch signaling in pancreatic cancer cells	[87]
TW-37 and ApoG2	Induces PAR-4 in pancreatic cancer and synergizes with gemcitabine	[84]
Apogossypolone	Suppression of Bcl-2 and Mcl-1 in pancreatic cancer	[90]
